# Declining incidence of Parkinson’s disease in Israel (2002–2021)

**DOI:** 10.1007/s00702-025-02984-2

**Published:** 2025-07-22

**Authors:** Yacov Balash, Tamar Zohar, Ronit Gilad, Anda Eilam, Amos D. Korczyn

**Affiliations:** 1 Department of Neurology, Kaplan Medical Center, 76100 Rehovot, Israel; 2 Data Research Center, Kaplan Medical Center, 76100 Rehovot, Israel; 3 The Hebrew University of Jerusalem, 91120 Jerusalem, Israel; 4 Departments of Neurology, Physiology and Pharmacology, Faculty of Medicine, Tel-Aviv University, 6997801 Tel-Aviv, Israel

**Keywords:** Parkinson's disease, Epidemiology, Time trend, Incidence, Average annual percent changes, Age-period analysis, Joinpoint regression

## Abstract

The results of investigations of the trends of the incidence of Parkinson’s disease (PD) over time in numerous developed countries showed that aging and increasing life expectancy are leading to an increase in both. We investigated the crude and age-adjusted incidence rates (AAIRs) of PD based upon registry data of Israel’s largest health maintenance organization between 2002 and 2021 according to joinpoint regression. We applied an age-period analysis to further identify patterns of AAIR changes, and calculated longitudinal age curves of PD rates (“local drift”) as well as annual change of the expected age-specific and expected age-adjusted AAIR (“net drift”). The overall AAIR of PD declined from 57 ± 1.0 to 20.3 ± 0.5 per 100,000 over 20 years, representing a 2.8-fold decrease. The PD incidence decreased more rapidly among females (average annual percent changes [AAPC] − 5.3%, 95% confidence interval [CI] − 6.0–4.6, *p* < 0.001) than among males (AAPC − 4.5, 95%CI − 5.3–3.7, *p* < 0.001). AAIRs peaked at 209.9 (CI: 193.0–228.5) per 100,000 at a median age of 77.5 vs. 374.9 (CI: 350.9–400.5) years in females and at a median age of 82.5 years in males. AAIRs gradually declined in males to 63.3 (CI: 52.2–84.1) per 100,000 and in females to 29.7 (CI: 21.4–41.1) per 100,000 for the 97.5-year-old group in both sexes. This first assessment of the trends of the incidence of PD in Israel documented its progressive decline from 2002 to 2021, especially among the very elderly. This decline may reflect refined diagnostic capabilities and enhanced health, quality of life and environmental conditions in Israel.

## Introduction

Parkinson’s disease (PD) is a common and disabling neurodegenerative disease of unknown etiology. Progressive dopamine deficiency, caused by degeneration of dopaminergic cells of the substantia nigra (Hornykiewicz 1966) and accumulation of misfolded alpha synuclein with Lewy bodies formation, is considered to be the underlying cause of PD (Spillantini et al. 1997; Jellinger 2003). The proportion of elderly people with PD is reportedly growing with the increase in life expectancy in recent decades, causing alarming predictions regarding the increase in the number of patients with PD inevitably resulting in a significant increase in the burden on medical services, social support, and society as a whole (Chen 2016; Dorsey et al. 2018; Savica et al. 2016; Wanneveich et al. 2018). In contrast, there are repeated reports of a decrease in the incidence of PD (Dammertz et al. 2023; Darweesh et al. 2016; Wong et al. 2019). Therefore, as Rocca (2018) noted, there is a great need for high-quality sources of data on the incidence of PD specific to individual countries and regions.

Israel is one of the developed countries with a constantly increasing population, having grown more than 16 times since its establishment in 1948. It has currently reached a population of 10.027 million people (Central Bureau of Statistics, December 2024) due to both an influx of immigrants of various ethnic and geographic origins who arrived from different regions of the world as well as to natural growth. Israel has an excellent medical system, and law mandates healthcare for all citizens.

We performed a population-based study of PD patients based upon data from the Clalit Health Service (CHS), the largest health maintenance organization in Israel, insuring more than four million persons. This population is not differed from that insured by other health services. We conducted a time-trend analysis of the incidence of PD, comparing patient sex distribution in different age groups. The aim of the current study was to determine the national trend in the incidence of PD over the past two decades and contribute the Israeli status to the pool of international data.

## Materials and methods

### Study design and data source

This register-based retrospective cohort study used a PD patient dataset, which was compiled by certified neurologists and family physicians who prescribed dopaminergic medications. The data were collected from the chief Clalit Research Data sharing platform powered by MDClone (https://www.mdclone.com) and based upon ICD-10 diagnostic codes. Anonymized information on age, sex, and dates of PD diagnosis, as well as the data concerning the population insured by CHS from 01/01/2002 to 12/31/2021 were extracted by the CHS Data Research Center at the Kaplan Medical Center (author TZ).

The patients were identified as having PD by two criteria: (1) being listed by the International Classification of Diseases, 10 Revision, clinical code for PD (G20) performed by neurologists of the CHS or included in medical records of the patients by other medical specialists both in hospital and outpatient settings; (2) having a minimum of two purchases of prescribed anticholinergic drugs, MAO-B inhibitors, dopamine agonists, levodopa, or amantadine. Identification of the second criterion was guided by the principles of constructing drug tracer algorithms developed earlier for the Israeli population at large (Chillag-Talmor et al. 2011). According to the relevant literature, fulfillment of these research criteria should ensure a positive predictive value as high as 86–94% for correct PD diagnosis in routinely collected healthcare datasets (Harding et al. 2019; Shumsky et al., 2009; Wei et al. 2016).

#### Statistical analysis

The collected data were entered into Microsoft Excel 2016 spreadsheets and then exported to the Fixlen, SEER*Prep^1^, and the SEER*Stat statistical programs (versions 1.8, 2.6.0 and 8.4.4, respectively). The above programs eventually allowed us to calculate the annual incidence rates and the age-adjusted incidence rates (AAIR) of PD with their confidence intervals (CI) per 100,000 of the all-age Israeli population by direct standardization to Segi’s world population standard (Ahmad et al. 2000). The data were analyzed by permutation tests (Kim et al. 2000) of the Joinpoint Regression Program (version 5.3.0.0) in order to determine the significance of changes in annual incidence rates as well as the AAIRs across successive calendar periods. Crude and AAIRs were calculated by dividing PD patients into 18 age groups from 0 to 85 + years according to the World Health Organization (WHO) (Ahmad et al. 2000). We estimated the significance of the average annual percent changes (AAPC) according to the t-criterion of the joinpoint regression (joined linear segments on a logarithmic scale) estimated by utilizing generalized linear models assuming a Poisson distribution (Clegg et al. 2009).

The age-period analysis^2^ is a parametric statistical method providing information about the effects of age and period on the trend in PD incidence rates. The data were assessed using the US National Cancer Institute A–P–C tool (https://analysistools.nci.nih.gov/apc/). The input data for this analysis were new PD cases and population counts divided into 12.5-year age groups (40–44…95–99), and into four 5-year periods (2002–2006, 2007–2011, 2012–2016, and 2017–2021) for males and for females. The default ages for reference were the means of each age group. The analysis functions included longitudinal age curve, period rate ratios (RRs), net drift, and local drifts. The longitudinal age curve depicted the fitted longitudinal age-specific rates adjusted for period deviations. Period RRs were the ratios of age-specific rates in each period relative to the reference period. Local drift represented annual percent change of the expected age-specific AAPC over time specific to age groups, while the net drift indicated the change of the expected age-adjusted rates over time. The two-tailed Wald’s χ^2^ test was used to test the null hypothesis (net drift = 0, local drifts = net drift, all period rate ratios = 1). The degrees of freedom (d.f.) counted the number of free parameters included in each test. Only *p*-values lower than 0.05 were considered as significant for all calculations.

## Results

The CHS registered 42,075 incident cases of PD during the study period, of whom 23,020 were males (54.7%). The average age of the patients at PD diagnosis was 75.5 ± 10.3 years.

### Overall trend

The crude incidence of PD showed a gradual decrease from 97.1 (95% CI: 94.05–100.5) per 100,000 during 2002 to 33.99 (95% CI: 32.36–35.7) per 100,000 during 2021, representing a 2.9-fold reduction (Table 1). The age-adjusted incidence of PD decreased from 57 ± 1 (95% CI: 55.1–59) per 100,000 during 2002 to 20.3 ± 0.5 (95% CI: 19.3–21.4) per 100,000 during 2021, representing a 2.8-fold reduction (AAPC − 4.8, 95% CI: − 5.5 to − 4.1, *p* < 0.001). In males, the AAIR fell from 71.6 ± 1.7 (95% CI: 68.3–75) to 26.9 ± 0.9 (95% CI: 25.1–28.7) per 100,000, representing a 2.7-fold decrease (AAPC − 4.5, 95% CI: − 5.3 to − 3.7, *p* < 0.001). In females, the AAIR decreased from 46.2 ± 1.2 (95% CI: 43.9–48.6) to 15.1 ± 0.6 (95% CI: 13.9–16.4) per 100,000, representing a 3.06-fold decrease (AAPC-5.3, 95% CI: − 6.0 to − 4.6, *p* < 0.001) for the same study period (Table 1).

**Table 1:** Total number of PD patients and insured persons and crude and age-adjusted PD incidence rates in Israel (2002–2021)

Year	Total annual number of incident PD patients	Total population insured by the CHS	Crude incidence rates (CI)	AAIR (CI)
2002	3585	3,689,223	97.17(94.05–100.5)	57.00 (55.1–59)
2003	3214	3,723,595	86.31(83.38–89.35)	50.07 (48.3–51.9)
2004	2726	3,733, 940	73.01(70.32–75.80)	42.36 (40.7–44.1)
2005	2408	3,755,410	64.12(61.61–66.73)	37.02 (35.4–38.6)
2006	2305	3,787,312	60.86(58.43–63.4)	34.96 (33.5–36.5)
2007	2238	3,823,193	58.54(56.16–61.01)	33.52 (32.1–35)
2008	2041	3,859,204	52.89(50.64–55.23)	30.69 (29.3–32.1)
2009	1983	3,902,303	50.82(48.63–53.1)	29.70 (28.3–31.1)
2010	2116	3,947,442	53.60(51.37–55.94)	30.98 (29.6–32.4)
2011	2049	4,012,145	51.07(48.91–53.33)	30.09 (28.7–31.5)
2012	2049	4,083,402	50.18(48.05–52.4)	29.81 (28.5–31.2)
2013	2034	4,165,755	48.83(46.75–50.99)	29.19 (27.9–30.6)
2014	1833	4,245,312	43.18(41.25–45.2)	26.54 (25.3–27.9)
2015	1795	4,317,562	41.57(39.7–43.54)	25.72 (24.5–27)
2016	1791	4,375,473	40.93(39.08–42.87)	25.79 (24.5–27.1)
2017	1673	4,450,095	37.59(35.48–39.44)	23.17 (20.0–24.4)
2018	1631	4,519,967	36.08(34.38–37.88)	22.42 (21.3–23.6)
2019	1582	4,572,050	34.60(32.94–36.35)	21.24 (20.2–22.4)
2020	1434	4,627,923	30.99(29.42–32.63)	18.88 (17.9–20)
2021	1588	4,672,254	33.99(32.36–35.7)	20.33 (19.3–21.4)

*AAIR* age-adjusted incidence rate per 100,000 populations, age-adjusted to the WHO 2000–2025 standard; *AAPC* average annual percent change, *CHS* Clalit Health Services, CI, 05% confidence intervals (lower and upper levels); *PD* Parkinson’s disease

### Trend by age groups

**Fig. 1:**
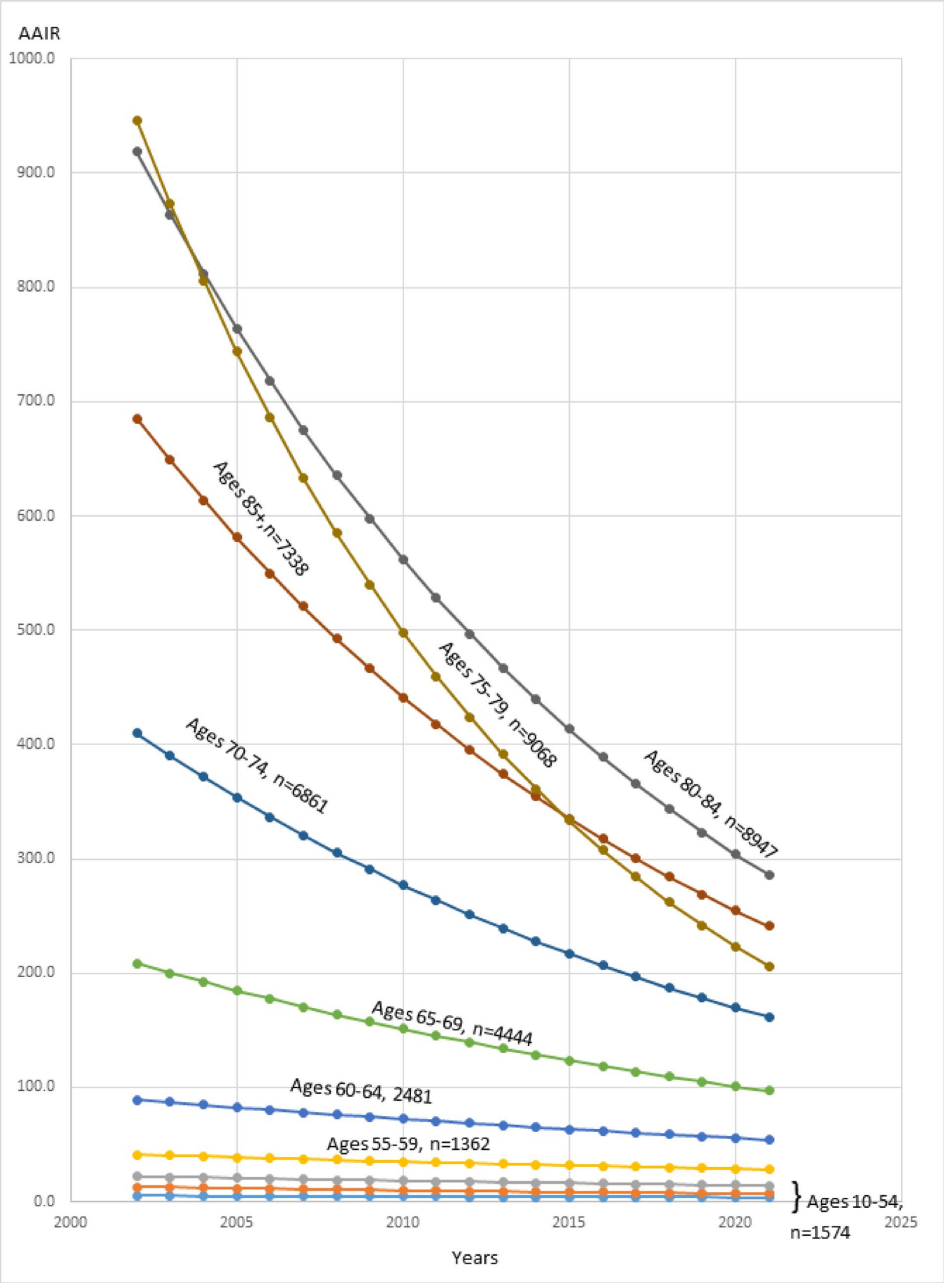
Age adjusted incidence rates (AAIR) in PD according tonumber of patients (n) in age groups in Israel from 2002 to 2021

Throughout the entire study period, all age groups showed a decreasing trend in AAIR, in which the steepest decline belonged to the oldest age group, 85 + years (AAPC − 7.7, 95% CI: − 8.5 to − 7.1, *p* < 0.001), while the lowest rates were calculated for the 40- to 44-year age group (AAPC–− 1.8, 95% CI: − 4.3 to − 0.8, *p* = 0.188) (Fig. 1). The decrease in PD AAIR became significant in males above the age of 55 years (AAPC − 2.4, 95% CI: − 3.6 to − 1.3, *p* < 0.001), and in females above the age of 60 years (AAPC-3.0, 95% CI: − 4.1 to − 1.9, *p* < 0.001).

### Analysis of the effects of age

**Fig. 2:**
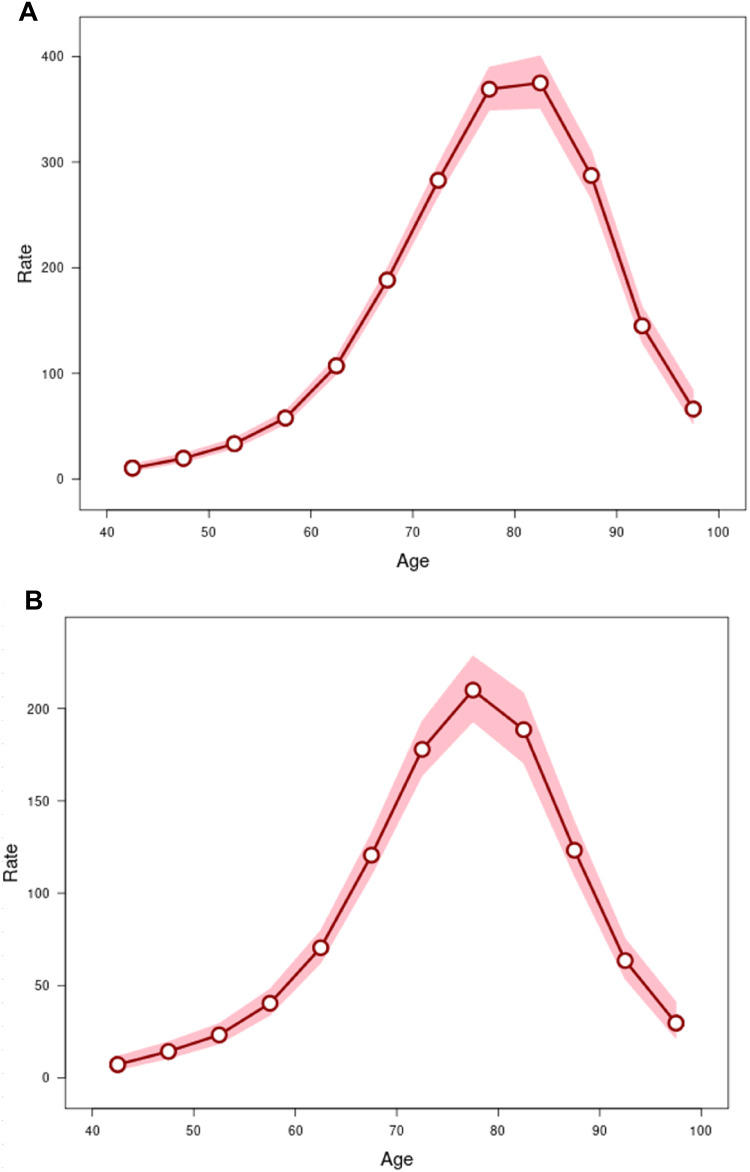
**A** Longitudinal age (years) curve of PD incidence rates with 95% CI (shaded area) in males. **B** Longitudinal age (years) curve of PD incidence rates with 95% CI (shaded area) in females

The effects of age were assessed by longitudinal age curves of PD onset rates according to sex (Fig. 2), which had an inverted U shape. The AAIR for the mean group age of 42.5 years were 10.3 (CI: 7.6–14.1) and 7.2 (CI: 4.5–11.8) per 100,000 in males and females, respectively (Table 2). They peaked to 374.9 (CI: 351.0-400.5) in males at the median age of 82.5 years per 100,000, and up to 210.0 (CI: 193.0-228.5) at a median age of 77.5 years in females. However, AAIR decreased in older people, and they were 66.3 (CI: 52.2–84.1) and 29.7 (CI: 21.4–41.1) per 100,000 at a mean age of 97.5 years in males and females, respectively. Local drifts with net drift, characterizing annual percentage changes in PD-onset ages, were highly significant in both sexes, thus rejecting the null hypothesis. Wald’s test F^2^ results were 138.1 in males and 69.8 in females (*p* < 0.00001) (Table 3).

**Table 2:** Age-group analysis of age-adjusted incidence rates of PD by sex according to Clalit health service data in Israel (2002–2021)

Males			Females	
	PD AAIR	95%CI	PD AAIR	95%CI
Age group, y (mean)				
40–44 (42.5)	10.3	7.6–14.1	7.2	4.5–11.8
45–49 (47.5)	19.5	15.9–24.0	14.3	10.5–19.6
50–54 (42.5)	33.4	28.9–38.7	23.3	18.4–29.4
55–59 (52.5)	57.8	51.8–64.4	40.4	34.0–48.0
60–64 (62.5)	107.1	99.1–115.8	70.5	62.2–79.7
65–69 (67.5)	188.4	177.3–200.0	120.6	109.8–132.4
70–74 (72.5)	282.8	268.3–298.1	177.9	163.8–193.2
75–79 (77.5)	369.0	349.3–389.8	210.0	193.0–228.5
80–84 (82.5)	374.9	351.0–400.5	188.6	170.6–208.5
85–89 (87.5)	287.3	265.5–311.0	123.2	109.4–138.7
90–94 (92.5)	145.0	128.9–163.0	63.5	53.6–75.3
95–99 (97.5)	66.3	52.2–84.1	29.7	21.4–41.1
Period, y				
2002–2006	1.35	1.30–1.41	1.31	1.23–1.39
2007–2011	1.00	1.00–1.00	1.00	1.00–1.00
2012–2016	0.92	0.88–0.96	0.83	0.78–0.89
2017–2021	0.69	0.65–0.72	0.63	0.59–0.68

*AAIR* age-adjusted incidence rate per 100,000 people; CI, 95% confidence interval

(lower and upper levels); *PD* Parkinson’s disease

**Table 3:** Local drifts with net drift according to the two-tailed wald’s χ^2^ test

	Males			Females		
Null hypothesis	χ^2^	d.f.	p-value	χ^2^	d.f.	*p*-value
Net drift = 0	520.6	1	< 0.00001	277.7	1	< 0.00001
All period RR = 1	571.8	3	< 0.00001	284.0	3	< 0.00001
All local drifts = net drift	138.1	12	< 0.00001	69.8	12	< 0.00001

*d.f.* degree of freedom, *PD AAIR* Parkinson’s disease age-adjusted incidence rates, *RR* rate ratios

CI, 95% confidence interval (shaded area); *PD* Parkinson’s disease

## Discussion

Reflecting the above-mentioned international trend, the incidence of PD in Israel has also progressively declined from 2002 to 2021, especially among the very elderly. The most important finding of the current analysis is the demonstration of a significant reduction of the PD incidence of individuals older than a mean age of 82.5 in males and of 77.5 in females according to longitudinal age curves of the age-period analysis (Fig. 2A and B). This was much more pronounced in older patients of both sexes (Fig. 1; Table 2). The decrease in PD incidence became significant in males above the age of 55 years and in females above the age of 60 years.

The finding of a decrease in the incidence of PD was first described by De Pedro-Cuesta and Stawiarz (1991). Those authors found a significant reduction (56%) in the incidences of Parkinsonism for the Rochester (Minn., USA) population aged 40–69 years during 1945–1966. A recent German study based upon nationwide outpatient claims and drug prescription data revealed a decrease of 23–28% in the annual age- and sex-standardized PD incidence in almost all regions of the country between 2013 and 2019 (Dammertz et al. 2023). A 4-year study conducted in Korea found that the incidence of PD decreased steadily from 35.4 per 100,000 person-years in 2012 to 33.3 per 100,000 person-years in 2015 (p for trend < 0.0001) (Han et al. 2019). The decreases in AAIR of PD similar to that found in our study were reported in Taiwan between 2001 and 2011 where the average age-standardized incidence of PD fell steadily from 35.3 per 100,000 in 2005 to 28.8 per 100,000 in 2011 (Liu et al. 2016). The Rotterdam prospective, population-based cohort study (Darweesh et al. 2016) also found that the incidence of parkinsonism in general, and that of PD in particular, remarkably decreased between 1990 and 2011, when the incidence rate ratio decreased by approximately 60% (0.39; 95% CI: 0.19–0.72). A similar trend was observed in Ontario, where the age- and sex-standardized incidence of parkinsonism decreased by 13.0% for mid/late-onset parkinsonism but remained unchanged for young-onset parkinsonism over the 18 years from 1996 to 2014 (Wong et al. 2019).

Thus, over the past 20 years, we, as reported in the above-mentioned studies, confirmed a significant decline in the incidence of PD in Israel as well. The decline of AAIR could not be explained solely by an increase in the CHS population since the number of registered cases of PD not only did not increase but contrarily, progressively declined (Table 1). These results may indicate that increased awareness about PD among healthcare professionals and the public, together with improved diagnostic capacities, could lead to more accurate clinical diagnosis of idiopathic PD vs. vascular, drug-induced, toxic alternatives of this syndrome, as well as parkinsonism plus syndromes or rare genetic diseases with extrapyramidal involvement. This could have also affected the decrease in the number of diagnosed PD cases according to the G20 code of the ICD-10. As shown by the study of Han et al. (2019), the rate of drug-induced parkinsonism cases has increased in parallel with a decrease in the incidence of PD.

Contrarily, a number of studies have shown that the prevalence of PD has actually increased over the past several decades (Savica et al. 2016; Ben Shlomo et al. 2024; Xu et al. 2024; Atterling Brolin et al. 2025). The trend towards the increase in the incidence and prevalence of PD because of aging was suggested as being so serious that the worldwide spread was considered a global problem (Ou et al. 2021). This has been attributed to the effect of better survival of PD patients thanks to contemporary medicine. Nerius et al. (2017) observed an effect of decreasing PD rates among octogenarians and nonagenarians. but explained this finding as being due to diagnostic uncertainty when extrapyramidal signs of PD as tremors were erroneously associated with Alzheimer’s disease and with misdiagnoses or decline of elderly people to seek medical care.

Historically, Gowers (1892) had already noted a lower incidence of PD after the age of 70 years among his own PD cases spanning ~ 40 years. Likewise, we now showed that the greatest reduction in PD incidence rates occurred in the oldest age group of patients. Several reasons might explain this reduction. One is the lower rate of referral of older adults to expert neurologists. Apathy, depressive disorder, and cognitive impairments typical of elderly patients with PD (del Carmen et al., 2015; Szymkowicz et al. 2023; Emre et al. 2014) may change their self-awareness (Maier and Prigatano 2017) when the current disease in the presence of other existing conditions will be considered by them as being less important. Chronic polyorganic morbidity usually requires the simultaneous administration of several kinds of medicines which increases the likelihood of developing side effects of therapy, including those associated with drug interaction, and such side effects can manifest in worsening mobility in elderly patients. With ageing, the metabolism decreases due to both a decrease in basal metabolic rate and a decrease in physical activity. This can affect the appetite of elderly patients and lead to insufficient or unbalanced nutrition with deficiency of vitamins and microelements, which eventually can be a factor in PD deterioration (Bianchi et al. 2023; Tosefsky et al. 2024). Thus, a visit to the doctor can become a challenge for elderly people who have barely retained the ability to ambulate, not to mention PD patients. Therefore, the influence of socioeconomic factors, such as diminished access to healthcare institutions and insufficient utilization of rehabilitation services (Agoriwo et al. 2024) on the observed trends in PD incidence, and especially among the elderly, could be considered pivotal. In Israel, however, these factors may be of minor importance, given that the number of expert neurologists and movement disorders clinics in Israel have increased since the over 20 years, and mandated medical services allow free access to expert clinicians, together with anti-Parkinsonian drugs being available at low cost.

Although the majority of older adults have reportedly expressed needing more help than they currently receive, such as in transportation, housing, food, household assistance, and medical and mental health care (Olomi et al. 2019), neither they nor their caregivers appear to sufficiently seek help for their unmet needs because of low expectations, resignation, refusal, or withdrawal (Walters et al. 2001).

Notably, a reduction in the incidence of disease in old age has been noted in other neurodegenerative diseases, including Alzheimer’s disease and Creutzfeldt-Jakob’s disease (Bugiani 2020; Denouel et al. 2023). While the reason for this has never been satisfactorily explained, one theoretical explanation might be that of a “survivors effect” such that some patients may also have age-dependent protective genes or were being exposed to yet unknown protective environmental or medical factors. These potential explanations warrant further exploration. Although ageing is considered a major risk factor for PD (Kesidou et al. 2023) due to age-related dysregulation of the immune system (immunosenescence) and as being responsible for the weakened response to novel antigens and increased susceptibility to infections, there is evidence for an attenuation in immunosenescence in PD, particularly for a reduction in senescent CD8 T lymphocytes, which may indicate a modification of the immunological response in the elderly (Kouli et al., 2022). It is interesting to note that several authors have suggested that the effects of aging on natural mortality stabilizes in very old age (Wachter and Finch 1997; Kinsella 2005; Robine and Paccaud 2005).

Our analysis once again highlighted differences between sexes in the longitudinal age curves, revealing a lower initial PD rate, a smaller rise and an earlier decline of the rates in all of the female age groups (Table 2; Fig. 2). This is consistent with most of the relevant studies published worldwide. The incidence of PD appears to be higher in males, with a male-to-female ratio of 1.5 (Moisan et al. 2016), although the ratio may be lower and continue to decrease in Far East populations (Kimura et al. 2002; Zirra et al. 2022), possibly due to the increasing life expectancy of females (Pavon et al. 2010).

A study on the global burden of PD in 2021 also found that the incidence of PD onset decreased with age (Luo et al. 2025), similar to our findings. According to those authors, that finding may be explained by the predominance of females among nonagenarians. However, our data do not support that explanation since males predominated in all of our age groups (Table 2). Hormonal fluctuations beginning at puberty and continuing until menopause are thought to be contributing factors to this sex-dependant difference. Exposure to certain environmental triggers, such as endocrine-disrupting chemicals found in some plastic products, flame-retardants, pesticides, and some other products of daily use, may also add to the observed sex differences in reported incidence rates in PD (Roy et al. 2009; Hatcher-Martin et al. 2012; Rossi et al. 2017; Xu et al. 2021; Paul and Ritz 2022; Song et al. 2023; Stein 2024; Costa et al. 2024; Qi et al. 2024).

## Limitations

The main limitations of this study is that we could not take into account and exclude the false-positive PD cases due to cerebrovascular disease, head trauma, and psychosis at the time of or before the diagnosis of PD. Although experienced clinical experts made the diagnosis of PD, they did not use the same criteria. Some of them registered the Hoehn and Yahr stages (H&Y) (Hoehn and Yahr 1967), others used the United Kingdom Parkinson’s disease society brain bank clinical diagnostic criteria (UKPDSBB) (Gibb and Lees 1988), or the Unified Parkinson’s disease rating scale (UPDRS) (Fahn and Elton 1987), or the Short Parkinson’s evaluation scale (SPES) developed by us in Israel (Rabey et al. 1997). In addition, due to the conditions of anonymity of this study, we did not have the opportunity to verify PD diagnosis in individual cases in an effort to prevent diagnostic and/or coding errors. Some data on associated factors, such as PD stage, family history, socioeconomic status, diagnostic tests, imaging studies, etc., that could be informative in explaining the trend towards a decrease of PD prevalence in Israel were unavailable or inadequate. Another limitation is that we included all patients with PD in one group. However, as had been recently shown, PD is a syndrome (Korczyn 2024), and our findings of a decreased incidence in old age may not apply to some of its variants. Minor errors may have occurred in the data extraction from the CHS Research platform or in the calculation of the population insured by the CHS, but we assume that inaccuracies in the coding of primary diagnosis of PD in searching for patients using electronic databases are unlikely.

## Conclusions

This study is the most comprehensive and extensive big data-based analysis of the trend towards reduction in PD incidence rates over the past 20 years in Israel. These findings paralleled the results of studies conducted in several other countries with highly developed medical capabilities. This trend can be at least partially explained by less medical care seeking among oldest olds individuals, primarily females. However, we believe that it may also be a result of reduced exposure to yet unknown risk factors of PD over time, as well as the results of refined diagnostic capabilities and improvements in environmental conditions, quality of life, and health in Israel. Despite the decline in the incidence of PD, the ageing of the population and the increasing life expectancy of patients with PD will continue to increase the burden on health and social care services.

In our study, we show for the first time the effect of age on this decline. The fact that this decline was much more pronounced in those above age 80, and that the effect became gradually more severe as age advanced was unsuspected and needs to be further studied and explained. It is unlikely that the effect is unique to Israel. The population of the country is very heterogeneous, and particularly the older generation were born in different countries and have immigrated to Israel in different ages, and were exposed to different environmental conditions for decades, in Europe and the Middle East or North Africa.

## Data Availability

The primary data for the findings of this study are unavailable due to the CHS restrictions and requirements for anonymity.

## Footnotes

1 SEER is the Surveillance Epidemiology and End Results research program representing a log linear Poisson regression tool, elaborated in the US National Cancer Institute (https://seer.cancer.gov/about).

2 In this study, the age-period (A-P) analysis was a part of the age-period-cohort (A-P-C) analysis. Here, the A-P-C is presented in abbreviated form because the cohort analysis will be presented in another paper.
